# Patterns of catch and trophic signatures illustrate diverse management requirements of coastal fisheries in Solomon Islands

**DOI:** 10.1007/s13280-021-01690-z

**Published:** 2022-02-12

**Authors:** Patrick Smallhorn-West, Jan van der Ploeg, Delvene Boso, Meshach Sukulu, Janet Leamae, Mathew Isihanua, Martin Jasper, Janet Saeni-Oeta, Margaret Batalofo, Grace Orirana, Alick Konamalefo, Jill Houma, Hampus Eriksson

**Affiliations:** 1 WorldFish, Unit 2, LKP Building, Mission Place, PO Box 438, Honiara, Solomon Islands; 2 ARC Centre of Excellence for Coral Reef Studies, James Cook University, 1 James Cook Drive, Townsville, QLD 4810 Australia; 3 Malaita Fisheries Division, Malaita Provincial Government, Auki, Malaita Solomon Islands; 4 Australian National Center for Ocean Resources and Security (ANCORS), University of Wollongong, Wollongong, NSW Australia

**Keywords:** Community-based marine management, Coral reef, Fisheries co-management, Malaita, Marine conservation

## Abstract

Coastal fisheries are a critical component of Pacific island food systems; they power village economies and provide nutritious aquatic foods. Many coastal women and men actively fishing in this region rely on multi-species fisheries, which given their extraordinary diversity are notoriously difficult to both characterize, and to manage. Understanding patterns of fishing, diversity of target species and drivers of these patterns can help define requirements for sustainable management and enhanced livelihoods. Here we use a 12-month data set of 8535 fishing trips undertaken by fishers across Malaita province, Solomon Islands, to create fisheries signatures for 13 communities based on the combination of two metrics; catch per unit effort (CPUE) and catch trophic levels. These signatures are in turn used as a framework for guiding suitable management recommendations in the context of community-based resource management. While a key proximate driver of these patterns was fishing gear (e.g. angling, nets or spearguns), market surveys and qualitative environmental information suggest that community fishing characteristics are coupled to local environmental features more than the market value of specific species they target. Our results demonstrate that even within a single island not all small-scale fisheries are equal, and effective management solutions ultimately depend on catering to the specific environmental characteristics around individual communities.

**Supplementary Information:**

The online version contains supplementary material available at 10.1007/s13280-021-01690-z.

## Introduction

The Coral Triangle is globally recognized for the exceptional biodiversity and socioeconomic value of its marine environment (e.g. Coral Triangle Initiative Secretariat 2009; Cohen and Steenbergen 2015). Within this region, coastal fish and other aquatic foods make important contributions to local food systems (King and Lambeth 2000; Farmery et al. 2020). Securing a sustainable supply of fish is therefore a regional priority to ensure continuing access to nutritious foods and sustainable livelihoods (Farmery et al. 2021).

Effective fisheries management is particularly challenging for coastal fishers targeting multi-species tropical fisheries, where difficulties are compounded by characteristics of both the fishers and the fishery (McClanahan et al. 2015). In the first instance, patterns of resource use and fishing effort are often unknown, which makes it difficult to provide details about the form and function of management (Costello et al. 2012; Pitcher and Cheung 2013). In the second, these problems are compounded by the high diversity of tropical fisheries, where captured species have vastly different life-history characteristics (McClanahan et al. 2015).

Since managing coastal tropical fisheries depends on characteristics of the resources as well as the people using them, it is now well recognized that those who are affected by management should be actively involved in the decision-making process (Johannes 2002; Berkes 2007). Community-based resource management (CBRM) is a process where natural resources are managed by, for and with local community involvement (Western and Wright 1994). CBRM has been highlighted as a key strategy for ensuring the sustainability of coastal marine resources in the Pacific (Govan et al. 2008), and is, for example, the primary strategy employed to secure ecologically sustainable fisheries in Solomon Islands (van der Ploeg et al. 2020a, b). This governance approach not only enables stakeholders to adapt management to their desired outcomes, but also to the prerequisite conditions of their surroundings, for which they are most aware. For example, a mangrove fishery will have vastly different management requirements than a fishery on a fringing coral reef. By the nature of being local, CBRM therefore needs to be an adaptive process that begins with the diagnosis of specific patterns of resource use (Govan 2009).

Not all coastal fisheries are the same, and characterizing a fishery and its potential drivers, or creating fisheries ‘signatures’ of prospective communities, helps tailor management requirement to specific contexts. Catch dependent data have been used in many fisheries monitoring programs to quantify catch per unit effort (CPUE), which, while acknowledging its limitations (e.g. Radovich 1976; Lorenzen et al. 2006; Petrere Jr and Giacomini 2010), can provide a useful measure of catch efficiency and acts as a proxy for fisheries status (Cohen et al. 2013), with higher CPUE typically indicating healthier stocks (within comparable systems and gear types) (Lorenzen et al. 2006; Castello et al. 2011). However, the high diversity of tropical ecosystems also creates key challenges in understanding patterns of fishing and their implications for sustainability, since catch statistics may differ across hundreds of species as well as between gear types (Humphries et al. 2019). Yet from a CBRM perspective being able to employ a simple and straightforward metric that can provide quick insights on the status of a fishery carries clear value. In ecology, trophic levels express where organisms tend to operate in their respective food webs, and for multi-species fisheries can therefore be used as a composite metric to group hundreds of species where individual assessments are impractical (Graham et al. 2017; Humphries et al. 2019). Trophic level not only acts as a proxy for the diversity of catch, but is also associated with both how species are caught (e.g. gear types) and their functional roles (Villéger et al. 2017). For example, high trophic level species are much more easily targeted using hook and line fishing methods than low trophic level species (Ahmed and Hambrey 2005). Determining both the peaks and ranges of trophic levels in catch composition therefore helps to elucidate specific patterns of fishing and their implications for CBRM.

**Fig. 1:**
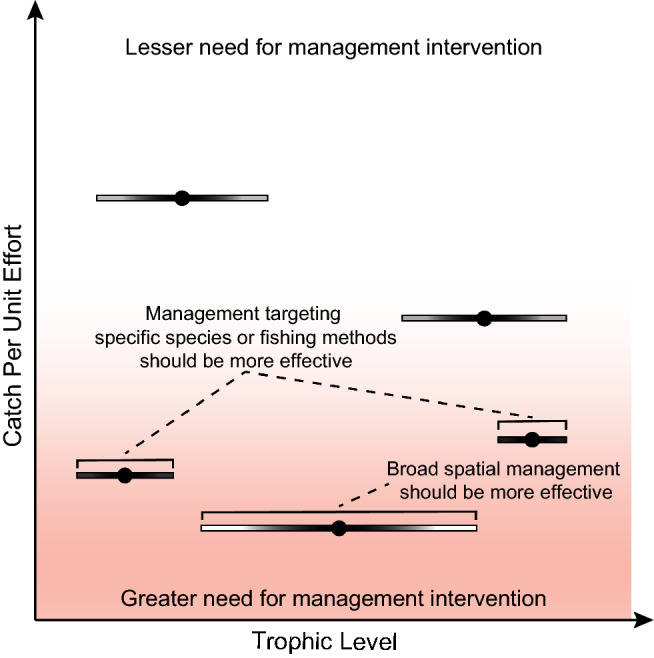
Conceptualization of the management implications from various patterns of catch per unit effort (CPUE) and trophic level of community catch composition. The color gradient indicates an increasing need for management interventions, while acknowledging the limitations of CPUE data for determining the status of a stock

Taken together, catch efficiency and trophic composition provide a simple signature that not only characterizes a communities fishery, but also generates specific management understanding. Figure 1 provides a conceptual diagram of the management implications from various configurations of CPUE and trophic signatures. CPUE signatures indicate the potential status of the stock and hence where management efforts should be focused (e.g. those with lower catch efficiency), while trophic signatures inform what kind of management is required. For example, if trophic levels indicate high degrees of specialization, then specific gear or species restrictions targeting those peaks should be the most effective management interventions. Conversely, if trophic levels are generalized, then spatial restrictions, such as periodically harvested closures or no-take reserves, will likely be more effective at improving overall sustainability.

Understanding the drivers of fishing patterns is also just as important for defining management options as the patterns themselves (Cinner et al. 2009). A key proximate driver of catch composition and efficiency is the type and size, and hence selectivity, of fishing gears employed (Wiyono et al. 2006; Ceyhan et al. 2010). However, while the type and size of gear used in fishing strongly influences what is captured, it will also be a product of what is available to catch, as well as the socio-environmental characteristics of a region and it’s fishery. Market forces and other socioeconomic factors have long been shown to play a key role in local patterns of fishing and catch (Brewer et al. 2013; Cinner et al. 2018; Cinner et al. 2020), as well as geographic and environmental conditions both at whole-of island and local community scales (Eriksson et al. 2018; Jouffray et al. 2019; Smallhorn-West et al. 2020; Russ et al. 2021). In market driven systems if specific species are overfished due to their high value then implementing species specific fishing restrictions or limits on their sale will be a better form of management over spatial restrictions. In contrast, these same restrictions will have little use in contexts where local environmental conditions limit the abundance of those species anyways. 

In this study, we use patterns of catch efficiency and trophic composition to generate specific “signatures” that characterize coastal fisheries across Malaita province, Solomon Islands. CBRM is a key priority of the Malaita Provincial Fisheries Office implementing the Fisheries Act (2015), which are actively working across Malaita to raise awareness and enable CBRM (Eriksson et al. 2020). In order to support these efforts, we address three research questions: (i) what are the patterns of catch across Malaita province? (ii) What drives these fisheries patterns? (iii) What are their management implications? We create unique fisheries signatures for 13 communities, which represents the first provincial level analysis of patterns of fishing for Malaita province. We then use a combination of provincial market data and qualitative environmental information to discuss potential drivers of village fishery characteristics. Lastly, we discuss how the diversity of fishing present within this multi-species fishery highlight the need for specific CBRM recommendations that are tailored for individual communities, and use the fishery signatures to provide a framework in which to do so.

## Methods

### Study location

Across Solomon Islands, coastal fisheries are an important contributor to food security and rural economies (Bell et al. 2009; Andersen et al. 2013; Arena et al. 2015), with roughly 40 000 households engaged in fishing, amounting to 37.5% of households in rural areas (Solomon Islands National Statistics Office 2011). However, fishing is generally a component of a diverse livelihood portfolio—relatively few rural households (2.5%) depend completely on fishing. Food for household consumption also comprises 65% of total household expenses (Faradatolo 2008), and most fish are consumed directly in the household or bartered or sold in the village. Malaita is the most populous (150 000) yet least developed province in Solomon Islands, comprising 27% of the total population while having the lowest human development index in the country (Sulu et al. 2015; van der Ploeg et al. 2020a, b). While a few specialized commercial fishers and traders do supply fish markets in Honiara and Auki with reef fish and shellfish, often sourced over relatively large distances, much of the reef area around Malaita province is considered underexploited (Brewer 2011; Sukulu et al. 2016). Approximately one third of villages in the Solomon Islands are currently employing community-based fisheries management based on either temporary spatial closures, species restrictions, or gear restriction (Brewer et al. 2021).

### Data collection

**Fig. 2:**
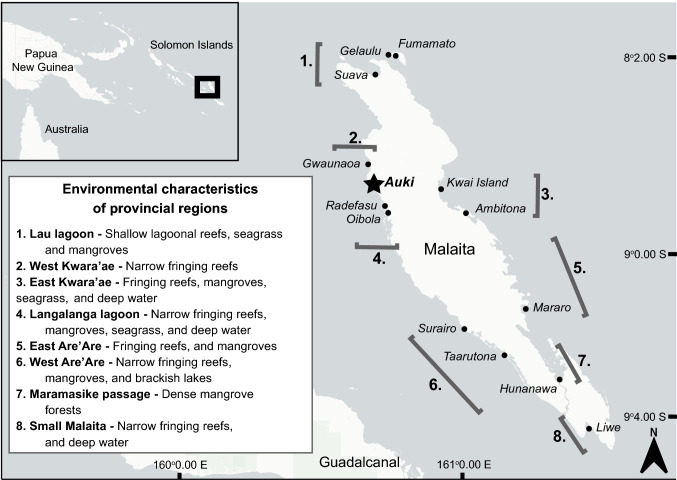
Map of the 13 study villages in Malaita province, Solomon Islands. The predominant environmental characteristics pertinent to the fisheries of each provincial region were determined during workshops between WorldFish and Provincial Department of Fisheries staff, many of whom were from the study villages

In 2017, local data collectors recorded information on coastal fisheries catches in 13 villages across Malaita province, with villages selected to cover the main littoral zones and fishing areas in the province (Fig. 2). One data collector from each village (except Mararo, *n* = 2) were trained and equipped with a booklet of standardized catch recording sheets to record landings twice a week for at least five fishers in their village, for 1 year. Data were collected opportunistically, but each data collector identified a way of working that best suited their local context (where, when, and how to collect the data). Data collectors were trained during a one week workshop and subsequently visited at intermittent intervals by WorldFish and/or Provincial Fisheries staff to improve coherency between sites and ensure data quality. In two instances (Oibola/Radefasu and Fumamato/Gelaulu) data collection occurred within two nearby areas that roughly belong to the same community cohort. In both cases, communities were kept separated due to different data collectors being employed in each community.

Data collection employed standard SPC creel survey methodology (FAME 2016) and for each trip recorded: date, departure time, arrival time, village name, number of fishers, weather, transport method (none, canoe, powered vessel), gear type (angling, netting or spearing), purpose (sale or food), number of each species caught and weight of each species caught. Mechanical weighing scales were used to record weight in 100 g intervals. Species were recorded in local names and subsequently identified using Moore and Colas (2016). Catch efficiency was calculated as CPUE (expressed in kg fisher h^−1^). Trophic levels for each species were downloaded from fishbase (www.fishbase.in), and if values for specific species were missing then similar species within the family were used instead. The mean trophic level for catch of each trip was then calculated based on the abundance of each fish species, with high values (> 3.5) indicating a catch of predominantly top predators, and low values (< 2.5) indicating a catch comprised herbivorous species. The mean trophic level of each trip was also calculated based on catch weight instead of abundance, with minimal differences to results (Fig. S1).

The data collection procedures introduced certain biases into the analysis. In most instances data collectors recorded the catch primarily of family and friends, and men are highly over represented in the data, with few fishing trips by women recorded (*n* = 608 trips). As a result of these limitations, this analysis could only investigate broad provincial patterns of catch efficiency for fish and not invertebrates, and was not able to examine patterns in total effort or yield.

### Drivers

To examine the influence of fish value on patterns of fishing, market surveys were conducted at the Auki fish market, the largest market in the province, over the same 12-month period as the village surveys (Sulu et al. 2018). The volume (abundance and weight), species, and value (price per kg) in Solomon Island Dollars (SID) of 100 042 fishes from 400 species, belonging to 47 fish families, were recorded to characterize the quantities and types of fish that pass through the Auki market to support management by the Malaita Provincial Fisheries Office. The relationships between species (i) value, (ii) proportion of total catch and, (iii) trophic level was then examined for all fish species recorded in both the market and village surveys.

Lastly, we also characterized the predominant habitats suitable for fishing around each village, along with other factors that could also strongly influencing patterns of catch (e.g. presence of a FAD, cultural preferences, etc.) based on personal observations and experiences of the authors.

### Analysis

Fishery ‘signatures’ were created for each community using density plots of CPUE and mean trophic level per trip and linear models were then used to compare between villages. Regression analysis was used to examine the overall relationship between median trophic level and CPUE across villages. We then examined gear type as a proximate driver for differences in village fishing characteristics. First, the proportion of trips conducted for each gear type (angling, netting and spearing) was calculated for each community. Then a linear mixed effect model was fit with CPUE as the response and trophic level and method as the predictor variables, with village and month included as random factors. Models were created with both fixed and random slopes and the one with the lowest AIC score selected. CPUE was log(*x* + 1) transformed and model fit was examined using partial residual plots. Finally, the correlation between the trophic level, value and the proportion of total catch of all 189 fish species present in both the village and market surveys was calculated using Pearson’s correlation coefficients.

## Results

**Fig. 3:**
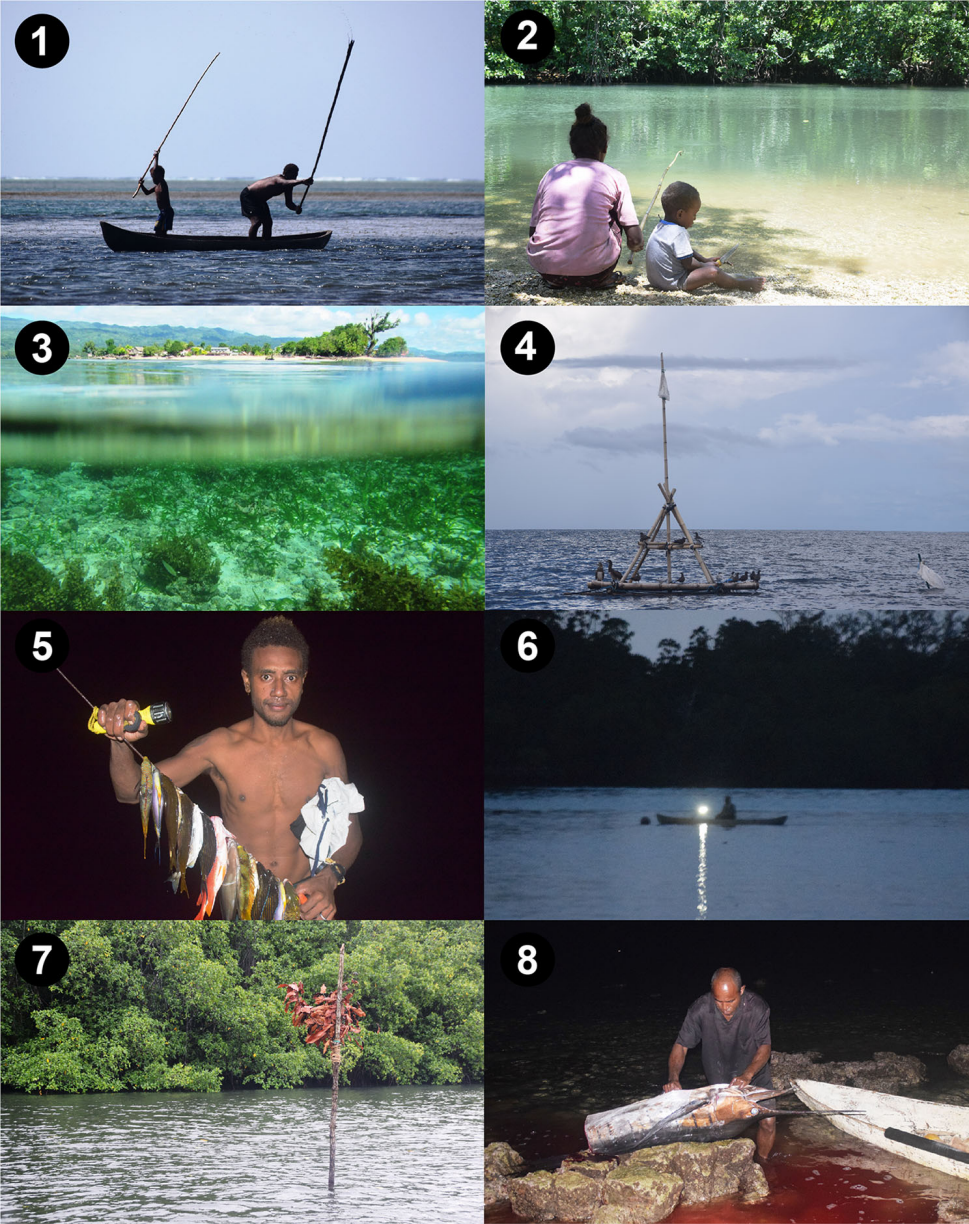
Diverse patterns of fishing in Malaita province, Solomon Islands. Each number corresponds to the regions in Fig. 1. **1** Fishing in Lau lagoon using sticks to chase fish towards set gillnets (*kwaesuru*). **2** Mother and child hook and line fishing in Gwaunaoa, West Kwara’ae. A major limitation of this study is insufficient data on womens fishing practices **3** Seagrass meadow in Kwai Island, East Kwara’ae. **4** Fish aggregating device (FAD) near Radefasu, Langalanga lagoon. **5** Spearfishing in Mararo, East Are’Are. **6** Nightfishing with light in West Are’Are. **7** Tabu marker in Hunanawa, Maramasike passage. **8** Sailfin processing in Liwe, Small Malaita. Photos by Jan van der Ploeg

Over the 12-month period a total of 8535 fishing trips were recorded from approximately 2000 individual fishers, yielding 189 223 fishes (50.2 tonnes) from 281 species (Fig. 3). The top five species caught (*Katsuwonus pelamis*, *Lethrinus olivaceous*, *Siganus fuscescens*, *Scarus ghobban and Lutjanus gibbus*) comprised 26% of total catch by weight, with *K. pelamis* catch comprising 11.3% of total catch (5.7 tonnes, Table 1).

**Table 1:** List of the top five species caught by weight for 13 villages in Malaita province, Solomon Islands. If data were not available from market surveys then the mean value of the family was used. No market data were available on *Triaenodon obesus* or *Herklotsichthys quadrimaculatus*. Percent total catch and cumulative % are by weight

Village	Species	Trophic level	Total weight (kg)	% Total catch	Cumulative %
Overall	*Katsuwonus pelamis*	4.03	5700.1	11.3	11.3
	*Lethrinus olivaceous*	4.2	3484.8	5.4	16.7
	*Siganus fuscescens*	2.28	1979.7	3.9	20.7
	*Scarus ghobban*	2	1715.3	3.4	24.1
	*Lutjanus gibbus*	3.63	1264.2	2.5	26.6
Ambitona					
	*Herklotsichthys quadrimaculatus*	3.27	1439.7	23.9	23.9
	*Euthynnus affinis*	4.47	550.1	9.1	33.0
	*Leptoscarus vaigiensis*	2.27	468.5	7.7	40.7
	*Naso hexacanthus*	3.06	290.2	4.8	45.5
	*Selar boops*	3.45	270.1	4.5	50.0
Fumamato					
	*Scarus ghobban*	2.00	682.1	13.4	13.4
	*Acanthurus olivaceus*	2.26	613.0	11.1	24.5
	*Lethrinus olivaceous*	4.20	565.9	10.3	34.8
	*Hipposcarus longiceps*	2.73	489.9	8.9	43.7
	*Siganus fuscescens*	2.28	345.2	6.3	50.0
Gelaulu					
	*Lethrinus olivaceous*	4.20	964.4	23.7	23.7
	*Siganus fuscescens*	2.28	557.4	13.7	37.3
	*Parupeneus ciliatus*	3.32	296.3	7.3	44.6
	*Acanthurus olivaceus*	2.26	293.2	7.2	51.8
	*Scarus ghobban*	2.00	200.3	4.9	56.7
Gwaunaoa					
	*Katsuwonus pelamis*	4.03	835.4	25.8	25.8
	*Lethrinus olivaceous*	4.20	169.4	5.2	31.0
	*Elagatis bipinnulata*	3.59	141.0	4.4	35.4
	*Euthynnus affinis*	4.47	137.4	4.2	39.6
	*Balistoides viridescens*	3.33	115.0	3.5	43.1
Hunanawa					
	*Liza vaigiensis*	2.18	264.5	13.8	13.8
	*Lutjanus bohar*	3.62	210.8	11.0	24.9
	*Epinephelus merra*	4.38	192.9	10.1	35.0
	*Caranx ignobilis*	4.48	184.9	9.7	44.6
	*Lutjanus rufolineatus*	3.80	178.6	9.3	54.0
Kwai Island					
	*Katsuwonus pelamis*	4.03	423.4	13.8	13.8
	*Thunnus albacares*	4.48	170.5	5.6	19.4
	*Sphyraena forsteri*	4.50	162.5	5.3	24.7
	*Coryphaena hippurus*	4.50	162.0	5.3	29.9
	*Scarus ghobban*	2.00	161.3	5.3	35.2
Liwe					
	*Katsuwonus pelamis*	4.03	1590.7	30.4	30.4
	*Elagatis bipinnulata*	3.59	546.6	10.4	40.8
	*Lethrinus olivaceus*	4.20	389.5	7.4	48.3
	*Euthynnus affinis*	4.47	301.5	5.8	54.0
	*Sphyraena forsteri*	4.50	298.2	5.7	59.7
Mararo					
	*Epinephelus merra*	4.38	462.6	6.5	6.5
	*Siganus fuscescens*	2.28	453.4	6.3	12.8
	*Lutjanus malabaricus*	4.09	407.0	5.7	18.5
	*Scomberomorus commerson*	4.38	365.9	5.1	23.6
	*Lethrinus olivaceous*	4.20	351.9	4.9	28.5
Oibola					
	*Katsuwonus pelamis*	4.03	176.6	18.7	18.7
	*Rastrelliger kanagurta*	3.19	129.7	13.8	32.5
	*Gerres oyena*	2.72	101.6	10.8	43.3
	*Caranx ignobilis*	4.48	62.2	6.6	49.9
	*Liza vaigiensis*	2.18	52.7	5.6	55.5
Radefasu					
	*Katsuwonus pelamis*	4.03	816.7	29.0	29.0
	*Coryphaena hippurus*	4.50	454.3	16.2	45.2
	*Decapterus macarellus*	3.24	171.3	6.1	51.3
	*Euthynnus affinis*	4.47	153.3	5.5	56.7
	*Sphyraena qenie*	4.50	134.5	4.8	61.5
Suava					
	*Rastrelliger kanagurta*	3.19	542.3	6.9	6.9
	*Euthynnus affinis*	4.47	512.6	6.6	13.5
	*Lethrinus olivaceous*	4.20	418.3	5.4	18.9
	*Siganus argenteus*	2.63	413.6	5.3	24.2
	*Lutjanus gibbus*	3.63	397.7	5.1	29.3
Surairo					
	*Katsuwonus pelamis*	4.03	1195.3	72.4	72.4
	*Aprion virescens*	3.98	77.6	4.7	77.1
	*Triaenodon obesus*	4.36	56.1	3.4	80.5
	*Thunnus albacares*	4.48	36.5	2.2	82.7
	*Herklotsichthys quadrimaculatus*	3.27	34.7	2.1	84.8
Ta’arutona					
	*Selar boops*	3.45	100.4	14.1	14.1
	*Caranx melampygus*	4.28	50.6	7.1	21.2
	*Sphyraena forsteri*	4.50	45.7	6.4	27.6
	*Decapterus macarellus*	3.24	42.5	6.0	33.6
	*Lutjanus bohar*	3.62	36.2	5.1	38.7

**Fig. 4:**
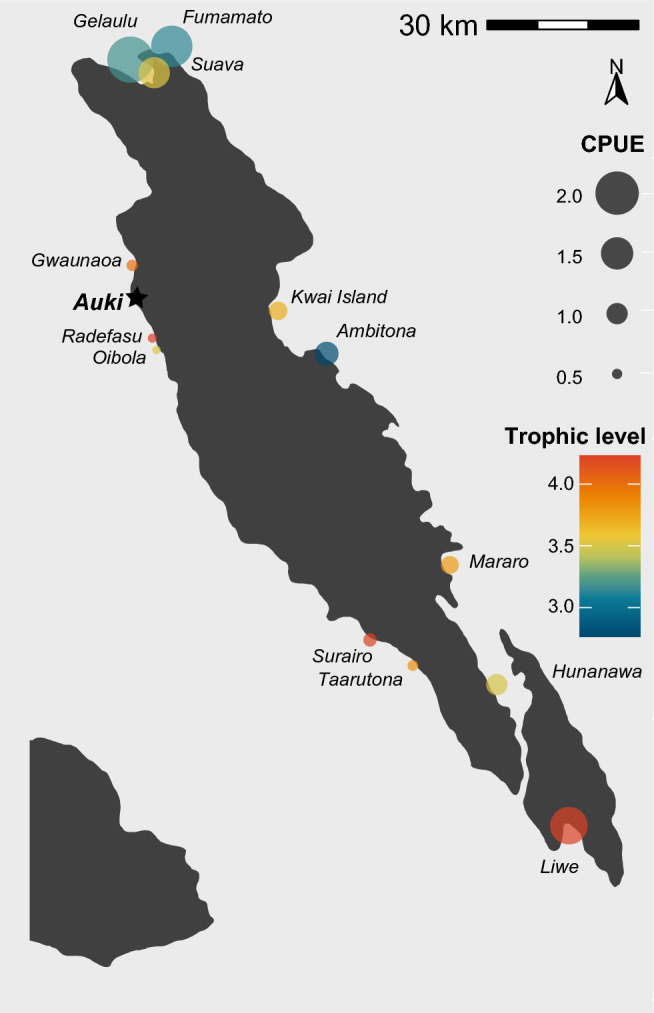
Map of median catch per unit effort (CPUE, kg fisher h^−1^) and mean trophic structure for 13 fishing villages in Malaita province, Solomon Islands. Trophic level values represent the mean values per catch. High trophic values (e.g. Liwe, 4.03) indicate a catch of predominantly top predators, and low values (e.g. Ambitona, 2.71) indicate a catch primarily comprised herbivorous species

Spatial patterns in fishing practices were evident across Malaita province (Fig. 4). Along the west coast CPUE was substantially lower across all villages (mean 0.52 kg fisher h^−1^) than anywhere else in the province. CPUE was greater for both the northern (Gelaulu, Fumamato and Suava, where communities comprise high proportions of professional fishers), and southern (Liwe), villages than those along either the eastern or western coastline. Conversely, the trophic signature of catches was generally highest along the western coast (mean 3.8), where catch was predominantly pelagics, and lowest in the northern villages (Gelaulu, Fumamato and Suava), where most fishes were reef or seagrass associated.

**Fig. 5:**
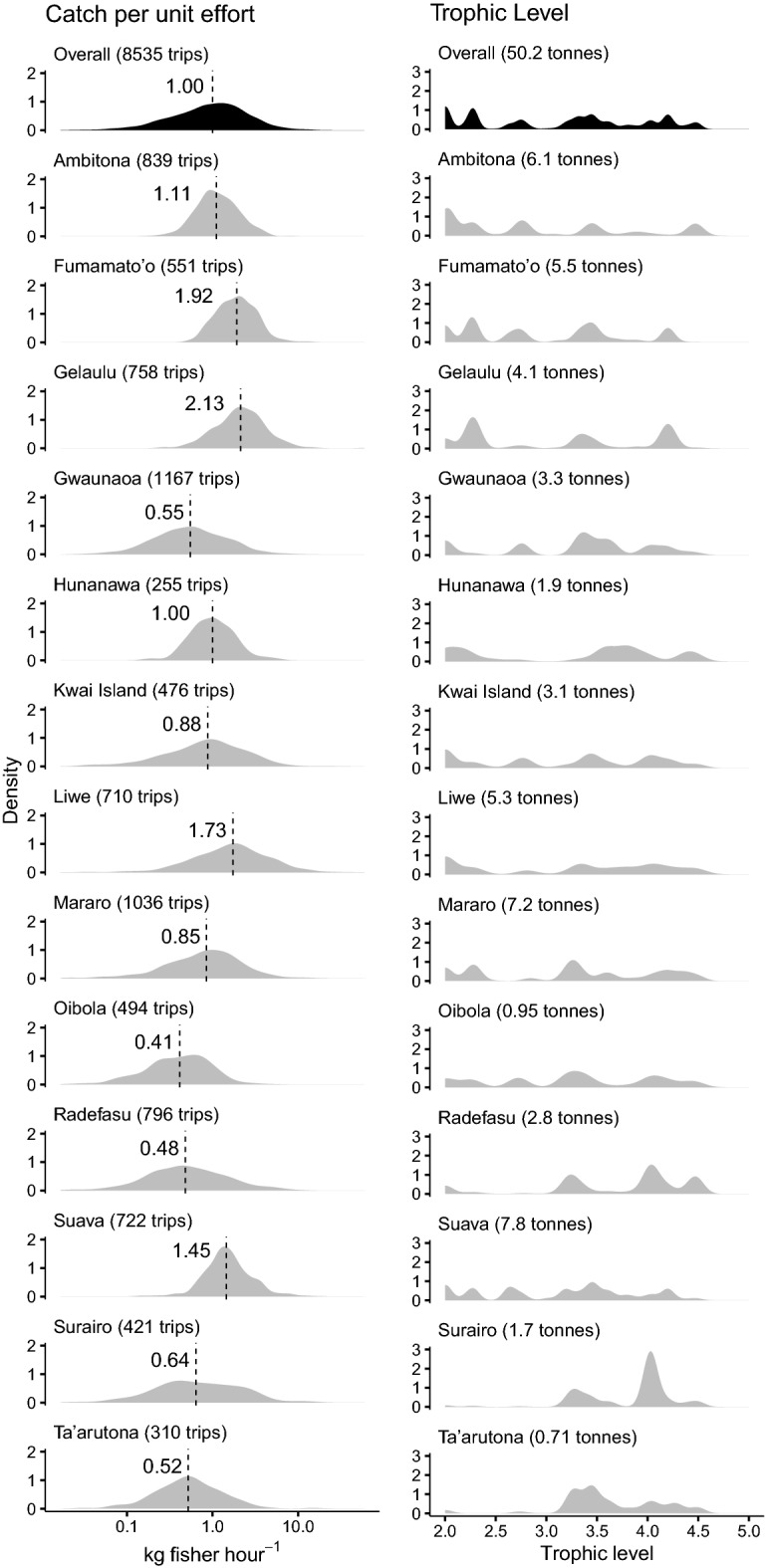
Catch efficiency and trophic ‘signatures’ for 13 communities in Malaita province, Solomon Islands. CPUE is on a log scale and dashed line represents median values. For CPUE the data represent the spread of values across all fishing trips from each community. For trophic level the data represent the spread of individuals caught across all fishing trips from each community. Values in parenthesis indicate the number of trips (CPUE) and total weight in metric tonnes (trophic level) for each community

The overall spread of CPUE data conformed to a similar distribution across all villages, with most trips having low catch efficiency and a large right skewed tail of higher efficiency trips (Fig. 5). Across all villages log transformed CPUE data therefore followed a normal distribution. Overall, the median provincial rate of catch efficiency was 1 kg of fish fisher h^−1^, but with significant variation between villages (Table S1). Median CPUE was greatest in Gelaulu, where it was more than double the overall median value (2.13 kg fisher h^−1^), and lowest in Oibola, where it was less than half the overall median value (0.41 kg fisher h^−1^).

**Fig. 6:**
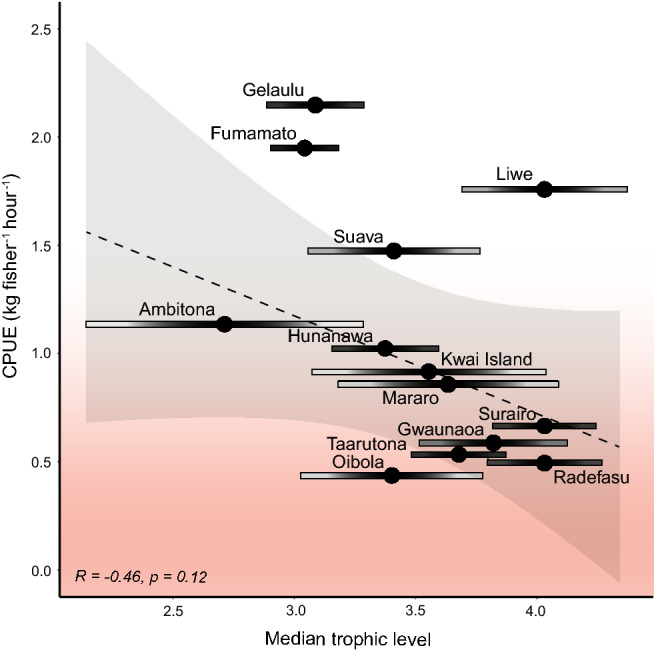
Relationship between trophic level and median catch per unit effort (CPUE) for 13 fishing communities studied in Malaita province, Solomon Islands. Color gradient towards lower levels of CPUE suggest where management could be prioritized. Trophic level bars represent variance around the mean, with the spread indicating the degree of fisheries specialization in each village, and hence whether management strategies should focus on restrictions of specific species or gears, or whether broad spatial approaches would be more suitable

The distribution of catch between trophic levels varied significantly between villages (Table S2), with some villages catching a wide range of trophic levels (e.g. herbivorous species through to top predators), while others had higher levels of specialization on species from specific levels, such as high level pelagics. Ambitona, Kwai Island, Mararo, and Oibola were the most generalized communities (i.e. those with the highest variance) (Table S3), with catch being roughly evenly distributed among trophic levels. Fumamato, Ta’arutona, Gelaulu, and Surairo were the most specialized villages, with Fumamato and Gelaulu specializing in low trophic level species (e.g. *S. ghobban*, *S. fusescens*), and Ta’arutona and Surairo specializing in high trophic level species (e.g. *K. pelamis*, *L. olivaceous*). With the exception of Liwe, all villages catching the highest trophic level species had median CPUE values below 1 kg fisher h^−1^ (Fig. 6). There was a moderate negative relationship between median values of CPUE and trophic level for each community, with villages specializing in high trophic level catches having approximately 50% lower CPUE on average, although given high variability and low sample size this trend was not significant.

**Fig. 7:**
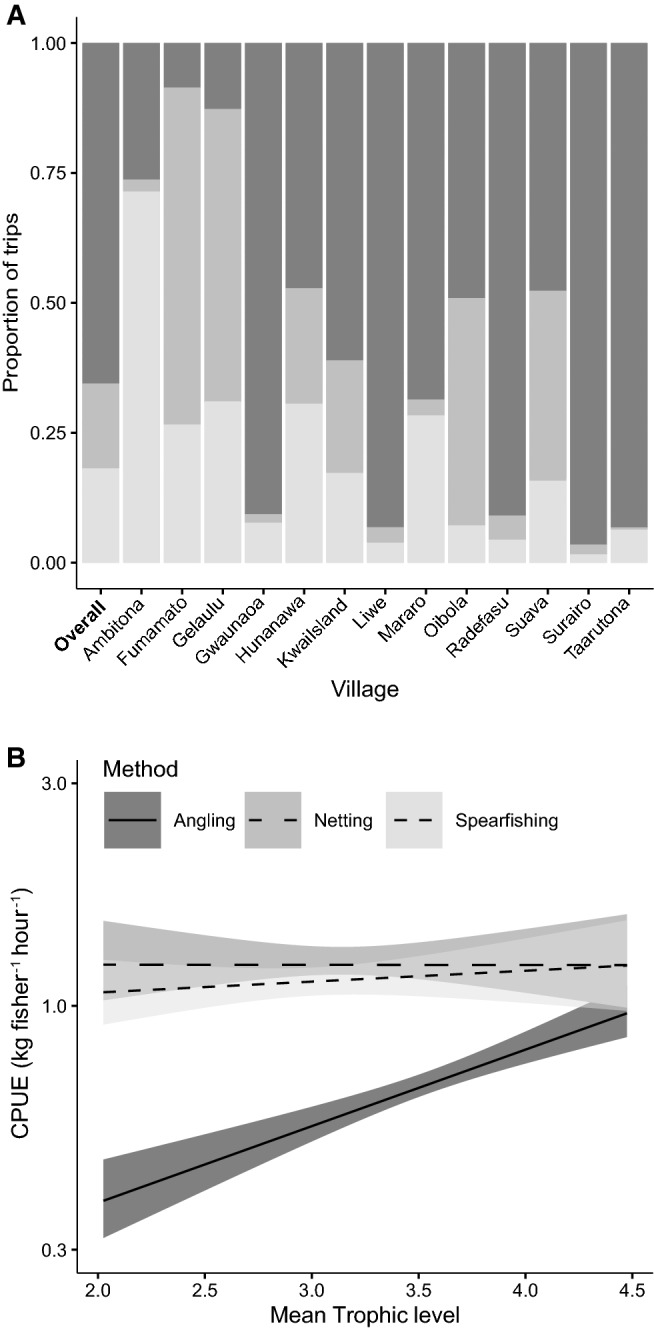
The proportion of fishing trips for each village based on fishing method (top), and the relationship between mean trophic level of catch and CPUE (kg fisher h^−1^) between fishing methods (bottom). Note that CPUE is on a log scale

There were substantial differences in the types of gear employed for fishing across Malaita province, as measured by proportion of trips by gear (Fig. 7; Table S4). The most common gear type overall was angling (65%), followed by spearfishing (18%) and netting (17%). This pattern was broadly similar within villages, with angling being the most common gear type in all but Ambitona, Fumamato, and Gelaulu. Villages with the highest trophic level catches (i.e. Gwaunaoa, Liwe, Radefasu, Surairo, and Ta’arutona) were intuitively also those with the greatest use of angling, whereas villages catching predominantly lower trophic levels (i.e. Ambitona, Fumamato, and Gelaulu) had a greater emphasis on spearing or netting. There was also a significant difference in the relationship between trophic level and CPUE between fishing methods, with angling having over twice the efficiency for top predators as for mid-range or low trophic level species. In general angling was less efficient than either netting or spearfishing, and only for high trophic level species did it approach a similar efficiency.

**Fig. 8:**
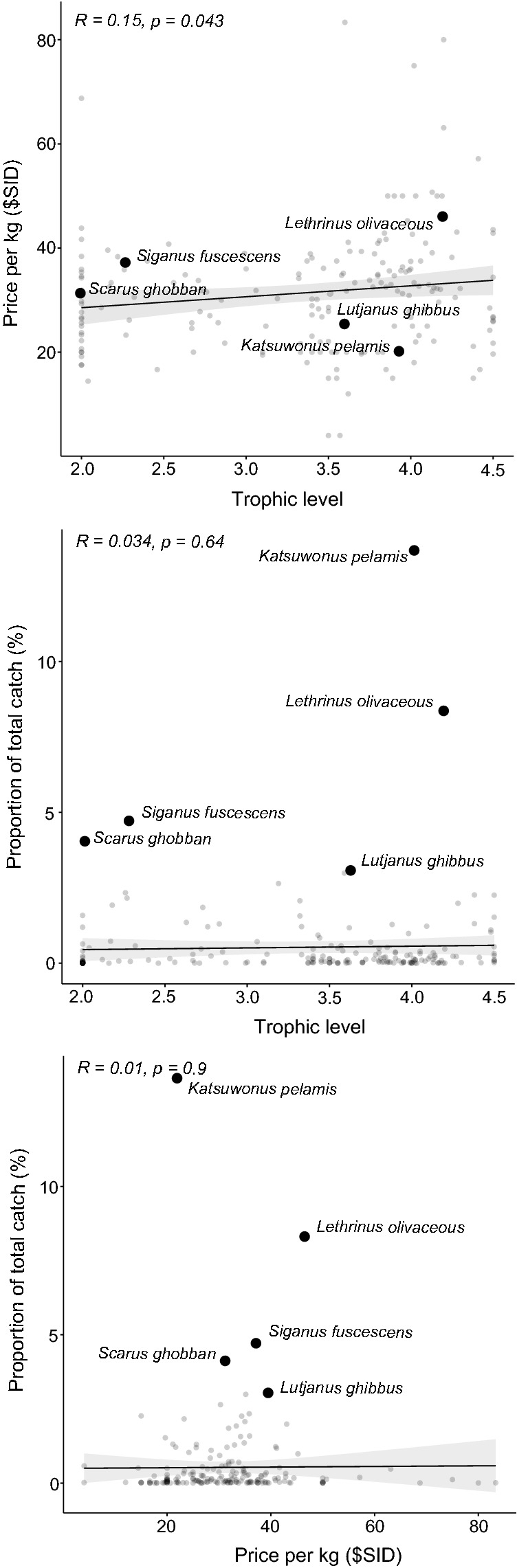
Relationships between trophic level, value, and proportion of total catch for the 189 fish species identified in both the Auki market surveys and village catch surveys in Malaita province, Solomon Islands. Each point represents one species. Value is represented as price per kilogram in Solomon Island Dollars ($SID). The five most abundant species included in the village catch data are noted

Overall, there was little evidence of any relationship between trophic level, market value, and proportion of catch for species caught in Malaita province (Fig. 8). Although statistically significant, there was only a very weak positive relationship between trophic level and mean value of fish species. Likewise, there was no relationship between either trophic level or market value and the proportion each species comprising the total catch. The most commonly caught species also were not closely related along either trophic level or market value ranges. However, there were substantial differences in the proportion of total catch attributable to a few key species, with 8 out of the 13 villages having 50% or more of their catch coming from the top five most abundant species caught in their village (Table 1). The most specialized community was Surairo, in which 72.4% of catch was of *K. pelamis.* Conversely, Gwaunaoa, Kwai Island, Mararo, Suava and Ta’arutona caught a wider range of species, and in both Mararo and Suava the most commonly caught species (*Epinephelus merra* and *Rastrelliger kanagurta*, respectively) comprised only 6% of the total catch. Lastly, Table 2 provides additional qualitative information collected during workshops between WorldFish and Provincial Department of Fisheries staff on patterns of catch and environmental characteristics of the fishing grounds surrounding each village included in this study.

**Table 2:** Additional qualitative information about patterns of fishing across Malaita province, Solomon Islands. This information was collected during workshops between WorldFish and Provincial Department of Fisheries staff

Village	Additional factors likely to influence patterns of fishing
Ambitona	Low population density. Seventh Day Adventist Church (SDA) limits harvesting of most marine invertebrates. A FAD was deployed in 2018 and has become a popular fishing spot—high use during data collection period. Many people employed by SDA with monthly salaries provided by the church.
Fumamato	Saltwata pipol (highly specialized local commercial fishers—sell fish to local market, or send coolers to Auki or Honiara) and densely populated village. FAD deployed but not a popular fishing spot (deployed by SPC cyclone PAM recovery project in 2017, but not part of local fishing methods). Locals primarily consume reef fish due to availability. However, pelagic species are preferred across Lau lagoon for ceremonial and traditional events (weddings, funerals, etc.). For special events fishers target tuna and snapper from lagoon, or large parrotfish, as these carry some prestige. Weekly community market trades at the mainland. Majority of fishers use fishing nets and most villagers rely heavily on sea resources for food and income. Very limited land for agriculture.
Gelaulu	Located very close to Fumamato with similar patterns of fishing.
Gwaunaoa	Densely populated but with few fishers. Harvested a lot of coral for lime production (Betel nut chewing). Very close to Auki market and supply with lime. Thus, their coral reefs are severely damaged, which is further exacerbated by storm surge. Most involved in subsistence farming. No FAD, but can fish FADs in nearby villages of Bio and Fote which have narrow fringing reefs.
Hunanawa	Densely populated community situated in Maramasike passage, which is an extensive mangrove area. While these mangrove habitats still currently remains intact, they are threatened by nearby logging operations. Since this village is located within mangrove habitat most catch is invertebrates, which was not included in this study. There is therefore a strong sampling bias for patterns of catch from this community.
Kwai-Island	Small, densely populated village of Saltwata pipol (commercial fishers). A FAD was recently deployed there but not during the time when the CPUE data were collected. Since people here live on an island they rely heavily on sea resources for food and income. Situated close to a large mainland market in Faumamanu, people from Kwai Island typically sell fish at this market to farmers and buy agricultural food from farmers in exchange.
Liwe	Village is located in a densely populated bay. Some fishers target commercial FADs, but there is no market close by so most fish is either for consumption or sold at very low prices within the community. Limited cash flow since they are far from other communities.
Mararo	Very small community on east coast. Logging activities in the region began during sampling and threaten their marine habitats. Many fishers from Mararo sell fish in the logging camp at a small market, which has increased the commercial sale of fish and cash flow. A marine management area has been established by Mararo in mangrove habitat to protect invertebrates.
Oibola	Densely populated, close to the urban center and in the past heavily overfished. The abundance of reef fish species is very low compare to other parts of Malaita and most excess catches are usually sold in the Auki market. Fishers are highly dependent on FADs for their current catch. Dynamite fishing was very common in the past across Langalanga, although not as common now. Mangrove ecosystem is threatened by firewood collection. Numerous artificial islands have also been established in the lagoon from materials collected through coral mining. Fishing is a key livelihood on these islands.
Radefasu	Similar patterns to Oibola. Have been continually fishing on FADs since 2014 and excess catch sold in Auki market. Villages along the west coast and within Langalanga lagoon have a strong cultural preference for reef fish over pelagic species for special events, although primary consumption is pelagic species due to their availability. Strong history of overexploitation and dynamite fishing.
Suava	Densely populated community of Saltwata pipol (commercial fishers), with most families involved in fishing. FADs were deployed as alternative fishing ground to reduce fishing pressure on reefs. Located close to a market at Malu’u sub-center and one of main supplier of fish to Malu’u market.
Surairo	A FAD was deployed close to the village and used heavily during the sampling period. Some of the most intact mangrove ecosystem in Malaita, although it is currently threatened by logging activity.
Ta’arutona	Low population density and a largely intact reef system. Established a marine management area on the reef with a FAD deployed nearby to relieve fishing pressure on their reefs. At night most fishing occurs on the passage outside the reef using lights. Very low fishing pressure on reefs.

## Discussion

Community-based management of coastal tropical fisheries is generally treated as a specific set of strategies (e.g. spatial, gear or species restrictions) that can be applied to achieve a specific set of objectives (e.g. improve sustainable yields, maintain biodiversity) (Govan 2009; Jupiter et al. 2014). However, our results show that even across a single province, patterns of fishing and catch are highly diverse, with many species being caught across different trophic levels with various levels of efficiency. Therefore achieving specific management objectives is not only dependent on the strategy employed but also the context under which it is implemented. In this discussion we begin by providing a methodology by which to apply specific management strategies based on CPUE and trophic signatures, and how this relates to previous work done on characterizing coral reef fisheries using catch efficiency and trophic level, followed by a discussion of our findings specific relevance to Malaita province, Solomon Islands.

In general, patterns of catch efficiency indicate the overall status of the fishery, while trophic patterns specify what the fishery is. Hence, CPUE signatures indicate the extent to which management is required, while trophic signatures indicate what kind of management is required. Both patterns exist on a spectrum and we acknowledge the limitations and assumptions associated with overreliance on CPUE to assess overall stock status for multi-species fisheries (e.g. Radovich 1976; Lorenzen et al. 2006; Petrere Jr. and Giacomini 2010). Nevertheless, fisheries with low or declining CPUE and peaks in their trophic signature (i.e. trophic specialization) are those for which specific restrictions on species or fishing gear would likely be more effective. For example, since Surairo had very low median CPUE (0.62), and was highly specialized on catching *K. pelamis* (trophic level 4.03), the management strategy most likely to be effective within this community (assuming management is desired) would be restrictions on the harvest of *K. pelamis*, or the hook and line methods employed to catch them. Likewise, since Gelaulu was highly efficient (median CPUE 2.2) at catching significant amount of *S. fuscescens* (trophic level 2.0), if CPUE was to subsequently decline in the future then placing restrictions on fishing nets should be an effective management intervention, since these are the primary method used to harvest this species. For fisheries with more diverse patterns of catch (e.g. spread out trophic signatures), specific species or gear restrictions are likely to be ineffective since these typically only target one or a few species, and instead spatial restrictions such as no-take reserves, periodically harvested closures, and access restrictions will be more useful for improving the sustainability of the fishery. For example, in Mararo (median CPUE 0.85), only 28.5% of catch came from the top five species, so creating spatial sanctuaries where multiple species can recover should be more effective than either limiting the harvest of any one of these species, or restricting fishing methods that are only effective at catching specific trophic levels.

In this manuscript we combine metrics of catch efficiency and trophic level in a management relevant context, but much research has previously emphasized the importance of these two metrics individually for understanding coral reef fisheries (Campbell and Pardede 2006; Cinner and McClanahan 2006; Campbell et al. 2014; Humphries et al. 2019). For example, Graham et al. (2017) recognized that trophic level and reef fish biomass are tightly related, and was able to demonstrate that fisheries for species within the top tiers of trophic levels will likely only be supported under lightly fished scenarios. Conversely, Roeger et al. (2016) demonstrated that harvest efficiency of low trophic level small coastal pelagics was two to five times greater than for many reef associated species. Together these findings support the negative relationship between trophic level and catch efficiency observed in our data. Furthermore, these patterns also have substantial implications for fisheries management—that is there may be much greater resilience to overharvest in low trophic level species. There may therefore be merit in revisiting the concept of ‘fishing down the food web’ (Pauly et al. 1998), such that the decline in species with increasing effort may be non-linear, resulting in greater resilience than previously supposed.

By using a combination of catch efficiency and trophic level signatures, we were also able to provide the first fisheries profile of catch in Malaita province, Solomon Islands. Broadly, patterns of catch along the west coast of the province can be characterized by a focus on line fishing for high trophic level species that congregate around FADs, in particular skipjack tuna (*K. pelamis*). Fishing on FADs for high trophic level species was generally inefficient, up to four times lower than catch efficiency for lower trophic level species in other parts of the province. In contrast, for fishing along the northern coast of the province around Gelaulu, Fumamato, and Suava, catch was dominated by more herbivorous species that are caught by net and spear, and were harvested with much greater efficiency. This contrasts to CPUE estimates in Timore-Leste, which were generally twice as high on FADs (2.17 kg fisher h^−1^) compared to reefs (1.21 kg fisher h^−1^) (Tilley et al. 2019). Good value for high trophic level species could have provided a plausible mechanism for the continued targeting of fish around the FADs, which would compensate fishers for their very low harvest efficiency. However, since there was no relationship between the value of fish caught and their trophic level, or their proportion within the total catch, we suggest that the environmental contexts and historical patterns of fishing around Malaita province play a greater role in driving patterns of fisher catch than price.

Market forces are known to be a key driver of global patterns in tropical fishery resources and management (e.g. Cinner et al. 2018; Cinner et al. 2020). However, at the scale of an individual province within the Solomon Islands, where fisheries are generally considered underexploited and population density is low, characterizing the drivers of catch may require a more nuanced approach than is generally available across larger scales. Along the west coast and in particular near the main fish market in Auki there is a history of overexploitation (Sulu et al. 2015), and reduced reef fish assemblages in this area may be why fishers are using FADs to target species with lower catch efficiency. But beyond this area, market forces are unlikely to be the dominant driver of catch patterns in the province. Instead, broad differences in the ecology and productivity of the environment surrounding each community is likely to underpin the many and diverse provincial fisheries. Indeed, many studies emphasize the overwhelming importance of biophysical predictors on structuring reef fish assemblages (e.g. Jouffray et al. 2019). For example, both coral cover (Russ et al. 2021) and habitat rugosity (Smallhorn-West et al. 2020) can be much stronger determinants of fish biomass in tropical assemblages than fishing pressure. The natural setting in which fishing occurs is therefore likely to either expand or narrow the potential opportunities for successful management interventions (Jouffray et al. 2019).

In-depth qualitative knowledge by the authors (representing WorldFish and Provincial Fisheries Officers) further suggested that cultural contexts may also play a role in patterns of catch, although these too are inextricably tied to environment. While somewhat simplistic, there were suggested differences in cultural preferences for fish across the province both for regular consumption and special events. These differences appeared driven by the availability of different types of fish, with rarer species having higher prestige. At the sites in north Malaita, there was a suggested preference for low trophic level reef fish for day to day consumption, but high trophic level pelagic species preferred for events like feasting, weddings and funerals. WorldFish deployed FADs along the north coast of Malaita, where the catch was predominantly reef fish. However, feedback from communities suggested that they hardly use the FADs because they prefer to fish using gillnets on the seagrass meadows, also showing that fishing practices and skills are attuned to local habitats and target species.

The concentration of exploitation and human influence around key areas such as the northwest coast of Malaita is in stark contrast to where management and CBRM implementation efforts are typically focused (van der Ploeg et al. 2020a, b). Most marine conservation and management projects in the Solomon Islands are clustered around a few localities that are still considered ‘pristine’ by international organizations (Cohen et al. 2012). These projects typically involve intense engagement with small communities near expansive and highly productive reefs. However, these situations are not representative of most coastal areas around the Solomon Islands, nor are they where the need for management is greatest (Gassner et al. 2019). By focusing management on remote areas which are not priorities for conservation or food security issues (i.e. not at risk compared to other areas), these management interventions risk being residual (van der Ploeg et al. 2020a, b), that is they will have little impact on wider development trajectories since there is limited overlap with areas of high resource use (Devillers et al. 2015; Pressey et al. 2017). CBRM should instead be emphasizing the management of areas where pressure is greater, and where there is correspondingly a greater need to effectively control coastal resource use (Sulu et al. 2015; Sukulu et al. 2016). The low catch efficiency observed in some villages in this study provide a clear indication of which parts of the country fisheries management could be most effective.

This study provides an important first step in defining patterns of catch and fisheries dynamics within Malaita province, but it also has several key shortcomings that limit the interpretation and applicability of the results. First, the data were heavily biased towards men and mens fishing activities, with only 608 of 8535 trips recorded for women. This analysis is therefore largely indicative of patterns in mens fishing, and in particular provides no information on gleaning or the catch of invertebrates—which we acknowledge are significant and crucial for rural livelihoods (e.g. Grantham et al. 2020; Tilley et al. 2021). Second, sampling effort was not standardized so that the number of fishing trips recorded, and who conducted them, was not reflective of overall fishing pressure or yield within each community. It was therefore not possible to calculate metrics such as total effort, or total catch, and these could not be compared between communities. Instead, catch efficiency was the best metrics that could be used to compare patterns of fishing across the province. Third, the prices used from surveys in the Auki fish market to calculate value may not be indicative of the exact value of those same fish in regional areas. While the value of fish in general does appear to decline with distance from Auki, so that fish are much cheaper to buy further away, this pattern does not appear to be dependent on which species are caught (personal communication). Therefore, despite changes in the value of fish across the province, it is still likely that the patterns are not species dependent. Lastly, while CPUE and trophic level are able to provide descriptive insights into patterns of fishing, in of themselves they do not indicate whether these patterns are sustainable, and more information is required so as to consider the range of additional factors that might influence sustainability. For example, trophic level as a metric lacks the nuance to distinguish between pelagic vs. reef associated species. Considering these limitations, our analysis therefore represents a first approximation of mens fishing patterns across the province, but with substantial data gaps in particular for womens fishing activities and estimates of total fishing pressure. This analysis also acts as an initial step towards creating in-depth descriptive fisheries profiles for Malaita province.

Effective management doesn’t just depend on specific strategies and the objectives they aim to achieve, but also the context in which they are implemented. Our research has shown that even while within a single province patterns of fishing can be highly diverse, yet simple metrics exist that indicate where and how management should be implemented. Scaling CBRM should therefore emphasise management requirements to the specific conditions, as well as the desired objectives, of communities, with particular focus on the more impacted regions where management is needed most.

## Supplementary Information

Below is the link to the electronic supplementary material.

Supplementary file1 (PDF 523 kb)
