# Supplementary material for: Patterns of catch and trophic signatures illustrate diverse management requirements of coastal fisheries in Solomon Islands
Source: Ambio. 2022 Feb 12;51(6):1504–19. doi: 10.1007/s13280-021-01690-z (PMC9005606; doi:10.1007/s13280-021-01690-z)
Supplement: Supplementary file 1 — Supplementary file1 (PDF 523 kb) [file 13280_2021_1690_MOESM1_ESM.pdf]

Supplementary materials for: Patterns of catch and trophic signatures illustrate diverse management requirements of coastal fisheries in Solomon Islands

Patrick Smallhorn-West[1,2]*, Jan van der Ploeg[1], Delvene Boso[1], Meshach Sukulu[1], Janet Leamae[1], Matthew Ishihanua[3], Martin Jasper[3],Janet Saeni-Oeta[1], Margaret Batalofo[1], Grace Orirana[1], Alick Konamalefo[3], Jill Houma[3], Hampus Eriksson[1,4]

[1] WorldFish, Honiara, Solomon Islands

[2] ARC Centre of Excellence for Coral Reef Studies, James Cook University, Australia

[3] Malaita Fisheries Division, Malaita Provincial Government, Solomon Islands

[4] Australian National Center for Ocean Resources and Security (ANCORS), University of Wollongong, NSW, Australia

***Corresponding author:** patrick.smallhornwest@jcu.edu.au; +61423412550

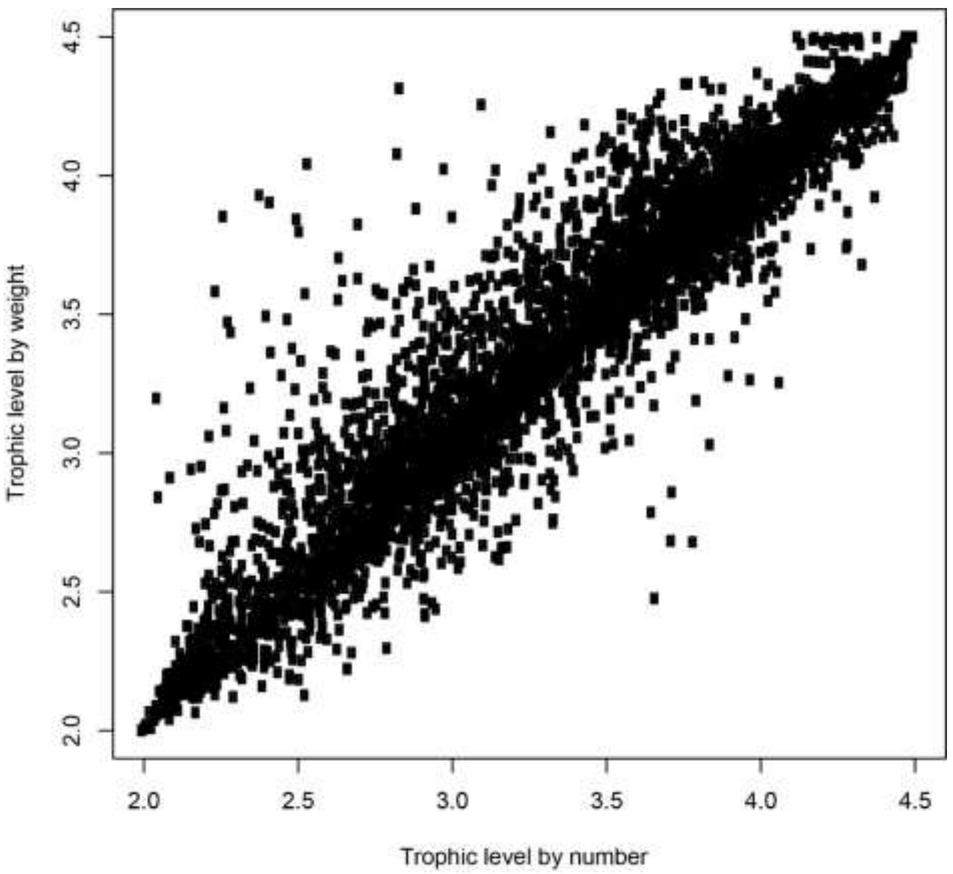

Figure S1. Relationship between trophic level calculations by number of fishes caught and weight of catch.

Table S1. Linear model output examining catch per unit effort (CPUE) across villages in Malaita village, Solomon Islands.

	log(CPUE + 1)		
Predictors	*Estimates*	*CI*	*p*
(Intercept)	0.76	0.75 – 0.77	**<0.001**
Village [Ambitona]	0.03	-0.01 – 0.07	0.119
Village [Fumamato]	0.33	0.28 – 0.38	**<0.001**
Village [Gelaulu]	0.42	0.38 – 0.46	**<0.001**
Village [Gwaunaoa]	-0.23	-0.26 – -0.20	**<0.001**
Village [Hunanawa]	-0.03	-0.09 – 0.03	0.387
Village [KwaiIsland]	-0.05	-0.10 – 0.00	0.066
Village [Liwe]	0.31	0.27 – 0.35	**<0.001**
Village [Mararo]	-0.07	-0.10 – -0.04	**<0.001**
Village [Oibola]	-0.36	-0.41 – -0.32	**<0.001**
Village [Radefasu]	-0.23	-0.27 – -0.20	**<0.001**
Village [Suava]	0.20	0.17 – 0.24	**<0.001**
Village [Surairo]	-0.11	-0.16 – -0.06	**<0.001**
Village [Taarutona]	-0.26	-0.31 – -0.20	**<0.001**
Observations	13606		
R^2 / R^2adjusted	0.119 / 0.118		

Table S2. Linear model output examining trophic level across villages in Malaita province, Solomon Islands.

	**nTroph**		
Predictors	*Estimates*	*CI*	*p*
(Intercept)	3.49	3.48 – 3.51	**<0.001**
Village [Ambitona]	-0.57	-0.62 – -0.51	**<0.001**
Village [Fumamato]	-0.42	-0.49 – -0.36	**<0.001**
Village [Gelaulu]	-0.30	-0.36 – -0.25	**<0.001**
Village [Gwaunaoa]	0.19	0.14 – 0.23	**<0.001**
Village [Hunanawa]	-0.11	-0.19 – -0.03	**0.005**
Village [KwaiIsland]	-0.03	-0.10 – 0.03	0.330
Village [Liwe]	0.34	0.29 – 0.39	**<0.001**
Village [Mararo]	-0.03	-0.07 – 0.02	0.220
Village [Oibola]	-0.09	-0.15 – -0.03	**0.003**
Village [Radefasu]	0.40	0.35 – 0.45	**<0.001**
Village [Suava]	-0.14	-0.18 – -0.09	**<0.001**
Village [Surairo]	0.32	0.26 – 0.39	**<0.001**
Village [Taarutona]	0.19	0.12 – 0.26	**<0.001**
Observations	13606		
R^2 / R^2adjusted	0.098 / 0.097		

Table S3. Variance in catch per unit effort and trophic level for each study village in Malaita province, Solomon Islands.

	**Variance**	
Village	*CPUE*	*Trophic level*
Ambitona	0.69	0.57
Fumamato	1.99	0.14
Gelaulu	10.56	0.20
Gwaunaoa	1.04	0.30
Hunanawa	0.73	0.22
KwaiIsland	2.86	0.48
Liwe	10.36	0.34
Mararo	3.10	0.45
Oibola	0.29	0.37
Radefasu	1.59	0.23
Suava	2.73	0.35
Surairo	3.24	0.21
Taarutona	1.82	0.19

Table S1. Model output for figure 7.

	log(CPUE)		
Predictors	*Estimates*	*CI*	*p*
(Intercept)	-1.73	-2.12 – -1.34	**<0.001**
nTroph	0.38	0.27 – 0.49	**<0.001**
Method [Netting]	1.93	1.46 – 2.41	**<0.001**
Method [Spearfishing]	1.69	1.28 – 2.09	**<0.001**
nTroph * Method [Netting]	-0.38	-0.52 – -0.24	**<0.001**
nTroph * Method [Spearfishing]	-0.32	-0.45 – -0.20	**<0.001**
**Random Effects**			
σ^2	1.00		
$\tau_{00\ Month}$	0.22		
$\tau_{11\ Month.nTroph}$	0.02		
ρ_{01}	-1.00		
ICC	0.01		
N $_{Month}$	12		
Observations	6803		
